# Poly(ethylene glycol)-modified silk fibroin membrane as a carrier for limbal epithelial stem cell transplantation in a rabbit LSCD model

**DOI:** 10.1186/s13287-017-0707-y

**Published:** 2017-11-07

**Authors:** Yijian Li, Yuli Yang, Lei Yang, Yuxiao Zeng, Xiaowei Gao, Haiwei Xu

**Affiliations:** 1 Southwest Hospital/Southwest Eye Hospital, Third Military Medical University, Chongqing, 400038 China; 2 Laboratory for Modern Silk, College of Textile and Clothing Engineering, Soochow University, Suzhou, 215123 China; 3 Department of Ophthalmology, 474 Hospital of the Chinese PLA, Xinjiang, Uyghur Autonomous Region 830013 China

**Keywords:** Silk fibroin membrane, Poly(ethylene glycol), Limbal epithelial stem cells, Culture methods, Limbal stem cell deficiency, Transplantation

## Abstract

**Background:**

Limbal epithelial stem cells (LESCs) play important roles in corneal epithelial homeostasis and regeneration, and damage to the limbus will lead to limbal stem cell deficiency (LSCD), with conjunctivalization and even visual impairment. Cultured LESCs have been used for ocular surface reconstruction, and silk fibroin (SF) membranes have shown potential as a substrate for LESC cultivation. Both culture methods and the carriers of LESCs affect outcomes following LESC transplantation.

**Methods:**

Rabbit LESCs were cultured from tissue explant, single cell-suspension, and cell cluster culture methods. Ratios of p63α and/or ABCB5-positive LESCs, differentiated corneal epithelial cells (CK12 staining), and corneal tight junction formation (Claudin-1 staining) were examined to choose the most applicable LESC cultures. SF membranes were prepared and modified by 400-Da poly(ethylene glycol) (PEG). The characteristics of stem cells and normal corneal differentiation of LESCs cultured on PEG-modified SF membranes were further examined by immunofluorescence staining and flow cytometric analysis. LESCs cultured on PEG-modified SF membranes (LESC/SF grafts) and PEG-modified SF membranes (SF grafts) were transplanted onto rabbit corneas with total LSCD. New blood vessels, corneal epithelial defects, and cornea clarity were examined after transplantation. Furthermore, corneal epithelial thickness, stromal thickness, and the percentage area of CK12-positive corneal epithelium were quantified 4 months after transplantation.

**Results:**

Tissue explant and single cell-suspension cultures harvested more p63α and/or ABCB5-positive LESCs, generated more CK12-positive corneal epithelial cells, and formed more corneal tight junctions than cell cluster cultures. Prepared PEG-modified SF membranes were transparent, flexible, and sturdy enough for surgical manipulation. LESCs cultured on PEG-modified SF membranes maintained characteristics of stem cells and normal corneal differentiation. LESC/SF grafts inhibited new blood vessels and rescued corneal epithelial defects in the rabbit total LSCD model. In addition, LESC/SF grafts repopulated the limbus and increased corneal epithelial thickness, stromal thickness, and the area percentage of CK12-positive corneal epithelium.

**Conclusions:**

LESCs from tissue explant and single cell-suspension cultures were more applicable corneal epithelial cells for ocular surface reconstruction. LESC/SF grafts repaired corneal epithelial defects and reversed LSCD, and PEG-modified SF membranes were suitable to be a carrier for LESC transplantation.

**Electronic supplementary material:**

The online version of this article (doi:10.1186/s13287-017-0707-y) contains supplementary material, which is available to authorized users.

## Background

The corneal epithelium consists of several stratified layers that are five or six cells thick. A complete corneal epithelial layer plays an important role in maintaining ocular surface stability and optical transparency. As the first protective barrier against the external environment, squamous epithelia are constantly renewed. Limbal epithelial stem cells (LESCs) in the limbus, the niche for LESCs, play important roles in corneal epithelial homeostasis and regeneration [1–4]. Several factors such as chemical (alkali/acid) or thermal injuries, ultraviolet and other ionizing radiation, and bacterial and viral infections can damage the corneal epithelial cells, including those of the limbal areas [5, 6]. In a severely injured cornea, the limbal and central corneal epithelia are both absent, resulting in conjunctival epithelial cells invading the corneal surface. The conjunctivalization, neovascularization, subepithelial scarring, and symblepharon formation severely affect the corneal transparency and even lead to visual impairment [6]. These diseases are called limbal stem cell deficiency (LSCD).

In ocular surface disorders with LSCD, the conventional treatments are donor healthy limbal tissue transplantation and keratoepithelioplasty [5, 7]. However, limbal epithelial allograft is limited by restricted donor sources and high risk of rejections. Advances in tissue engineering techniques offer another alternative treatment: cultured LESC transplantation [8–11]. LESCs were cultured and amplified, and the biological characterizations were identified by specific monoclonal antibodies, which lay a foundation for the clinical application of LESCs. These cultured LESCs have shown satisfactory effects in treating LSCD [8, 11]. Furthermore, using the air-lifting (AL) method, these cultured LESCs formed sheets which were similar to corneal epithelium in vivo [12, 13].

Nowadays, there are three isolation methods to culture LESCs. The tissue explant culture is the simplest way, in which LESCs migrate from the small limbal tissue biopsy samples, while the single cell-suspension cultures, method isolates LESCs using conventional enzymatic digestion with dispase. More recently, a new method was reported in which collagenase was used to digest deeply underlying stromal cells [14–16]. A cluster of cells was generated and seeded, so the method was named cell cluster cultures. Despite many reports of the three culture methods [16–18], a comprehensive comparison among these three culture methods is needed to prepare the most suitable LESCs for further clinical applications.

Silk fibroin (SF) is a group of fibrous proteins produced by some members of the classes *Insecta* and *Arachnida*, generally to make webs or cocoons. SF is a promising biomaterial owing to a variety of factors including its wide availability, relatively low cost, and proven biocompatibility [19, 20]. In ocular tissue reconstruction, SF membranes possess potential advantages over some other natural and synthetic biomaterials. SF membranes are transparent, flexible, permeable to solutes, and should promote adequate levels of cell attachment and growth. In addition, SF has a number of functional groups that can be reacted with molecules of interest (e.g., RGD sequences), and SF has been combined with those sequences found in extracellular matrix (ECM) molecules [21]. Furthermore, SF-based materials display strength and the rate of degradation can be controlled [20]. SF membranes and SF membranes modified with poly(ethylene glycol) (PEG) have shown significant potential as a substrate for corneal epithelial cell growth, and may therefore provide a suitable substitute for donor amniotic membrane (AM) in LESC transplantations [19, 22–24]. However, it will be necessary to ensure that these cultured LESCs on SF membranes maintain characteristics of stem cells and the ability to divide and differentiate into normal corneal epithelium both in vitro and following transplantation onto the ocular surface.

In this study, we firstly comprehensively compared the three cultures (tissue explant, single cell-suspension, and cell cluster cultures) to choose the most applicable LESC expansion method in vitro for ocular surface reconstruction. Furthermore, we examined whether PEG-modified SF membranes were suitable to be a carrier for LESC cultivation and transplantation.

## Methods

### Tissue explant cultures of rabbit LESCs

Primary LESCs were cultured from rabbits’ limbal tissue explants using a modification of protocol described previously [25]. In brief, an incision was made at the conjunctiva of the eye 3 mm behind the limbus and dissected toward the limbus and into the cornea up to 1 mm. After the conjunctiva was excised out at the limbus just behind the pigmented line (the palisades of Vogt), the limbal ring tissue was cut into 18–20 pieces of 1–2 mm^2^. These pieces were directly put onto six-well culture plates. After 16 hours of incubation in a drop of fetal bovine serum (FBS; Gibco), limbal tissue explants were grown in DMEM/F12 (1:1) medium (Hyclone), supplemented with 10% FBS, 10 ng/ml epidermal growth factor (PeproTech), 0.4 mg/ml hydrocortisone (Sigma), 0.1 nM cholera toxin A subunit (Sigma), insulin–transferrin–selenium (Gibco), 0.18 mM adenine (Sigma), 2 nM of 3,3,5-triiodo-l-thyronine (Sigma), and 50 IU/ml penicillin–streptomycin at 37 °C. The media were changed every 2 days. The cultures without visible fibroblastic and stromal outgrowth were used for future studies.

### Single cell-suspension cultures of rabbit LESCs

For single cell-suspension cultures, previously described protocols were used [10, 26]. The limbal ring tissues were cut into 10 pieces 4–5 mm in length and incubated at 37 °C for 18 hours with 2 IU/ml dispase (Roche). Limbal epithelium sheets were then isolated from limbal stroma and digested once again with 0.25% trypsin/1 mM EDTA (Invitrogen) for 5 minutes to isolate into single cells. The limbal epithelium single cell suspension, including stem cells, was seeded at a density of 5 × 10^3^ cells/cm^2^ into six-well culture plates which were precoated with fibronectin (Akron Biotech) for at least 30 minutes.

### Cell cluster cultures of rabbit LESCs

Primary LESCs from cell cluster cultures were cultured using protocols described previously [14–16]. The limbal ring tissues were cut into 10 pieces 4–5 mm in length and directly digested with 1 mg/ml collagenase (Sigma) in keratinocyte serum-free medium (KSFM; Gibco) for 18 hours to generate a cell aggregate termed ‘cell cluster’. The limbal clusters, including stem cells and stromal cells, were directly seeded into six-well culture plates which were precoated with fibronectin for at least 30 minutes. For the aforementioned cluster, single cells were obtained by further digestion with 0.25% trypsin/1 mM EDTA at 37 °C for 5 minutes to measure the colony forming efficiency (CFE).

### Histology, immunofluorescence, and hematoxylin and eosin staining

Rabbit ocular tissues and LESCs cultured on PEG-modified SF membranes were fixed in 4% paraformaldehyde (PFA), incubated in 30% sucrose, embedded in Tissue-Tek OCT compound, and mounted as 10-μm-thick sections on microscope slides. For immunofluorescence staining, cells or tissue sections on glass slides were fixed in 4% PFA and permeabilized with 0.3% Triton X-100. After being blocked in 10% secondary serum plus 3% bovine serum albumin (BSA) for 1 hour, cells or tissue sections were followed by an overnight incubation (16 hours) in primary antibodies at 4 °C. After washes, the cells or sections were incubated in appropriate secondary antibody for 1 hour at 37 °C. Cell nuclei were counterstained with DAPI (Beyotime, China). The following antibodies were used: rabbit anti-p63α monoclonal antibody (ab124762; Abcam), mouse anti-ABCB5 monoclonal antibody (ab140667; Abcam), rabbit anti-α-SMA polyclonal antibody (BM0002; Boster), mouse anti-Ki67 monoclonal antibody (550609; BD BioSciences), goat anti-CK12 polyclonal antibody (sc-17098; Santa Cruz), rabbit anti-CK12 monoclonal antibody (ab185627; Abcam), mouse anti-CK7 antibody (ab9021; Abcam), mouse anti-BrdU monoclonal antibody (ab1893; Abcam), rabbit anti-Claudin-1 polyclonal antibody (ab15098; Abcam), mouse anti-CD68 monoclonal antibody (MCA341R; AbD Serotec), and mouse anti-CD31 monoclonal antibody (ab9498; Abcam). The secondary antibody, Alexa Fluor-488 or 568-conjugated anti-mouse or rabbit immunoglobulin G (IgG) (Invitrogen), was used at a dilution of 1:600. For hematoxylin and eosin (HE) staining, tissue sections were incubated in hematoxylin for 5 minutes, followed by incubation in eosin for 2 minutes. Following several washes, the slides were then coverslipped in antifade mounting media (Beyotime). Pictures were taken with a fluorescence microscope (BX51; Olympus) or confocal microscope (FV1000; Olympus), and analyzed using ImageJ software.

### Flow cytometric analysis

To gain a quantitative analysis, including coexpression analysis, flow cytometry was performed on BD FACSCalibur (Becton Dickinson) according to the manufacturer’s instructions. In brief, cultured rabbit LESCs were fixed in 4% PFA and permeabilized with 0.3% Triton X-100. After blocking in 10% goat serum plus 3% BSA for 30 minutes, LESCs were followed by 60-minute incubation in primary antibodies at room temperature. After several washes, LESCs were followed by 45 minutes of incubation in secondary antibodies. For ABCB5 and p63α coexpression analysis, cells were incubated with ABCB5/p63α antibodies, and counterstained with goat anti-mouse AlexaFluor-488/647-conjugated IgG (Invitrogen). Dual-color flow cytometry was performed by acquisition of fluorescence emission at the Fl1 (AlexaFluor-488) and Fl4 (AlexaFluor-647) spectra.

### Colony forming efficiency

Rabbit LESCs (200 cells/cm^2^) from tissue explant, single cell-suspension, and cell cluster cultures were seeded onto fibronectin precoated six-well culture plates. To assess the CFE, colonies on six-well culture plates were fixed after 9–12 days of culture. After washes with PBS, the colonies were stained with crystal violet solution (Sigma-Aldrich). The calculation of CFE was based on the number of colonies divided by the number of LESCs seeded in six-well culture plates. The previous criteria and method were adopted to define the three clone types [27]. Holoclones were defined as large round colonies with smooth and regular borders and formed entirely by small cells with scarce cytoplasm. Meroclones were defined as large colonies formed by small cells but showing irregular borders and/or with areas containing large cells. Paraclones were defined as small colonies with wrinkled and irregular borders and formed by large cells. Finally, we compared CFE of each clone of LESCs from the three cultures.

### Preparation of PEG-modified SF membranes

Silk fibroin membranes were prepared as described previously with a modification [23, 24, 28]. Briefly, aqueous solutions of silk fibroin (4% w/v) were prepared from *Bombyx mori* cocoons in ethanol and used within 1 month. The mixture was made up of 4% w/v SF solution and 40% w/v PEG (molecular weight, 400-Da) solution with a ratio of 100:1. The SF/PEG solution was cast onto 12-well culture plates (200 μl per well) after intensive mixing. The ethanol and water in the solution were gradually evaporated and formed membranes in the 12-well cuture plates.

### Scanning electron microscopy

To investigate the morphology, PEG-modified SF membranes were dried overnight, sputter-coated with 10 nm gold, and observed using scanning electron microscopy (SEM; Zeiss).

### Culture of LESCs on PEG-modified SF membranes

The PEG-modified SF membranes were cut into circular pieces approximately 16 mm in diameter. The PEG-modified SF membranes were sterilized by submersion in 75% ethanol for 60 minutes, and stored then in PBS or culture medium for at least 12 hours. The resulting PEG-modified SF membranes were inserted into sterile 12-well plates for LESC culture. Primary LESCs from tissue explant cultures (P0) were digested with 0.25% trypsin/1 mM EDTA (Invitrogen) for 2 minutes, and then these cells including stromal cells detached from the plates were aspirated out. The LESCs that still adhered on plates were further digested with 0.25% trypsin/1 mM EDTA for another 5 minutes. These LESCs were seeded onto the PEG-modified SF membranes at a density of 3 × 10^4^ cells/cm^2^ and allowed to attach for 24 hours. The cells were cultured for 10 days with medium changes every 3 days. These LESCs cultured on PEG-modified SF membranes were used for transplantation, or fixed by 4% PFA for immunohistochemical staining.

### BrdU chase experiments

LESCs were cultured with fresh DMED/F12 medium containing 10 μM BrdU (Sigma) to label all of the cells that were dividing. After labeling with BrdU for 1 day continually, the cultures were chased for 10 days by switching to BrdU-free DMEM/F12 medium. The chase period was calibrated to dilute BrdU label from most cells but not any slow-cycling cells (which include putative quiescent stem cells). At 0, 4, and 10 days, LESC samples in triplicate were fixed in cold methanol at 4 °C for 10 minutes. All samples were incubated with 2 mol/L HCl at 37 °C for 45 minutes to denature DNA and neutralized in boric acid (pH 8.5) for 20 minutes. Incorporated BrdU-retaining LESCs were detected by immunofluorescent staining.

### Rabbit LSCD model

The New Zealand white rabbits (2–2.5 kg) were anesthetized by auricular vein injection of 3% pentobarbital sodium. The right eyes of rabbits were used to create the LSCD model and following transplantation. Drops of 0.5% proparacaine and 1% atropine were instilled onto the cornea at the beginning of the procedure. The whole limbus was removed to create a superficial keratectomy from 2–3 mm inside the limbus at a depth of approximately 200 μm with a round beveled corneal microblade. The remaining corneal epithelium was scraped off (total LSCD model) or not (limbus-deficient model) from the central cornea with a number 15 scalpel blade. Antibiotics (levofloxacin) and steroids (betamethasone) were applied to eyes immediately after the surgeries, and were administered three times a day for 2 weeks. All eyes underwent fluorescein sodium staining and slit lamp examination. The successful rabbit total LSCD model was used for further transplantations.

### Optical coherence tomography

All rabbits were scanned with Fourier-domain optical coherence tomography (FD-OCT) (Optovue RTVue-100 Fourier-Domain Optical Coherence Tomography System; OPTOVUE Co., USA). The wavelength of FD-OCT is 1310 nm, hence it can accurately evaluate the depth of the corneal lesion. All rabbits were scanned with FD-OCT at the peripheral 6 mm of the cornea before and after surgeries. Four radial lines at the peripheral cornea were measured and the following parameters were obtained: maximum depth of the pathology; and the corneal thickness corresponding to the maximum depth of the pathology.

### Cell label and LESC transplantation

Transplantations were performed on the rabbit corneas with LSCD. All rabbits were randomly assigned to each experimental group. The right eyes of each rabbit in all groups underwent 360° conjunctival peritomy followed by removal of the fibrovascular pannus. The SF grafts underwent PEG-modified SF membrane transplantation alone without expanded LESCs. The LESC/SF grafts underwent transplantation similar to contact lens-based cell delivery [29, 30], with LESCs cultured on PEG-modified SF membranes. Before the LESC/SF graft transplantation, LESC/SF grafts were labeled with DiO (D275; Thermo Fisher) for 30 minutes to trace these donor LESCs. The normal eyes were used as positive control, with the LSCD model (no grafts) as negative control. The PEG-modified SF membranes in SF and LESC/SF grafts groups were secured by interrupted episcleral 10–0 nylon monofilament sutures through the edge of the membranes to the conjunctiva. After transplantations, each eye underwent slit lamp examination and photography at 20, 30, and 60 days. All of these eyes underwent fluorescein sodium staining and slit lamp examination. Antibiotics (levofloxacin) and steroids (betamethasone) were applied to eyes immediately after the transplantation, and were administered three times a day for 2 weeks.

Consequent scorings of corneal neovascularization and corneal clarity were done in four quadrants of each eye. The degree of corneal neovascularization was scored as follows: grade 0, no neovascularization; grade 1, blood vessels reach less than one-third of the distance between the limbus and corneal center; grade 2, blood vessels reach between one and two-thirds of the distance between the limbus and corneal center; grade 3, blood vessels reach more than two-thirds of the distance between the limbus and corneal center; and grade 4, blood vessels reach the corneal center. The degree of corneal clarity was scored as follows: grade 0, severely dense opacity completely obscuring the pupil; grade 1, moderately dense opacity partially obscuring the pupil; grade 2, mild haze of minimal density; grade 3, trace or faint corneal haze; and grade 4, totally clear.

### Statistical analysis

The data were presented as the mean ± standard deviation (SD). Statistical analysis was performed using Student’s *t* test or one-way analysis of variance (ANOVA). *P* < 0.05 was considered statistically significant.

## Results

### Cultivation of rabbit LESCs from the three culture methods

**Fig. 1:**
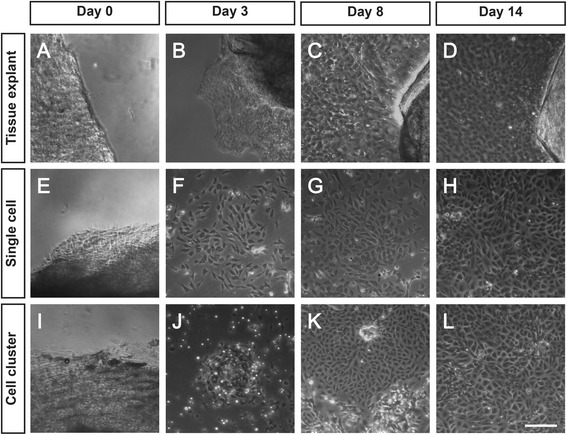
Rabbit LESCs cultured from the three culture methods. **a** Tissue explants on the plate. **b** Corneal epithelial-like cells migrated from tissue explants after 3 days of culture. **c**, **d** Corneal epithelial-like cells near the tissue explants. **e** Tissue explants became loose after digestion with dispase for 18 hours. **f**–**h** Small epithelial-like cells extended, fused, and generated epithelial sheet. **i**, **j** Tissue explants were subjected to collagenase digestion in KSFM for 18 hours to yield cell clusters. **k**, **l** Clusters grew like colonies with LESCs in the middle. Scale bar, 50 μm

To harvest the best LESCs for transplantation, we first compared the three cultures (tissue explant, single cell-suspension, and cell cluster cultures) to determine the most applicable LESC expansion method in vitro. In tissue explant cultures, the corneal epithelial-like cells migrated out from the limbal fragments in 2–5 days (Fig. 1a, b). The corneal epithelial-like cells around the tissue explants were more compact and uniform than peripheral cells. By 8 days, the corneal epithelial-like cells almost covered the whole culture plates (Fig. 1c). The corneal epithelial-like cells reached confluence and became an epithelial sheet in 2–3 weeks (Fig. 1d).

In single cell-suspension cultures, small epithelial-like cell colonies including about 10–20 cells were observed after 3–8 days of culture (Fig. 1e–g). By 8–10 days, the colonies began to fuse. The cells covered the whole culture plates and reached confluence by 10–14 days (Fig. 1h).

In cell cluster cultures, collagenase digestion yielded a loose cell aggregate (cell cluster). These cell clusters contained about 20–50 cells and proliferated very fast (Fig. 1i, j). By 8–10 days, these cell clusters grew like colonies. Consistent with previous reports [14, 15], LESCs were also in the middle of the cultures and were surrounded by peripheral stromal cells (Fig. 1k). By 14 days, these LESCs in the middle of the cultures expanded over more area (Fig. 1k, l).

### Characterization of rabbit LESCs from the three cultures

**Fig. 2:**
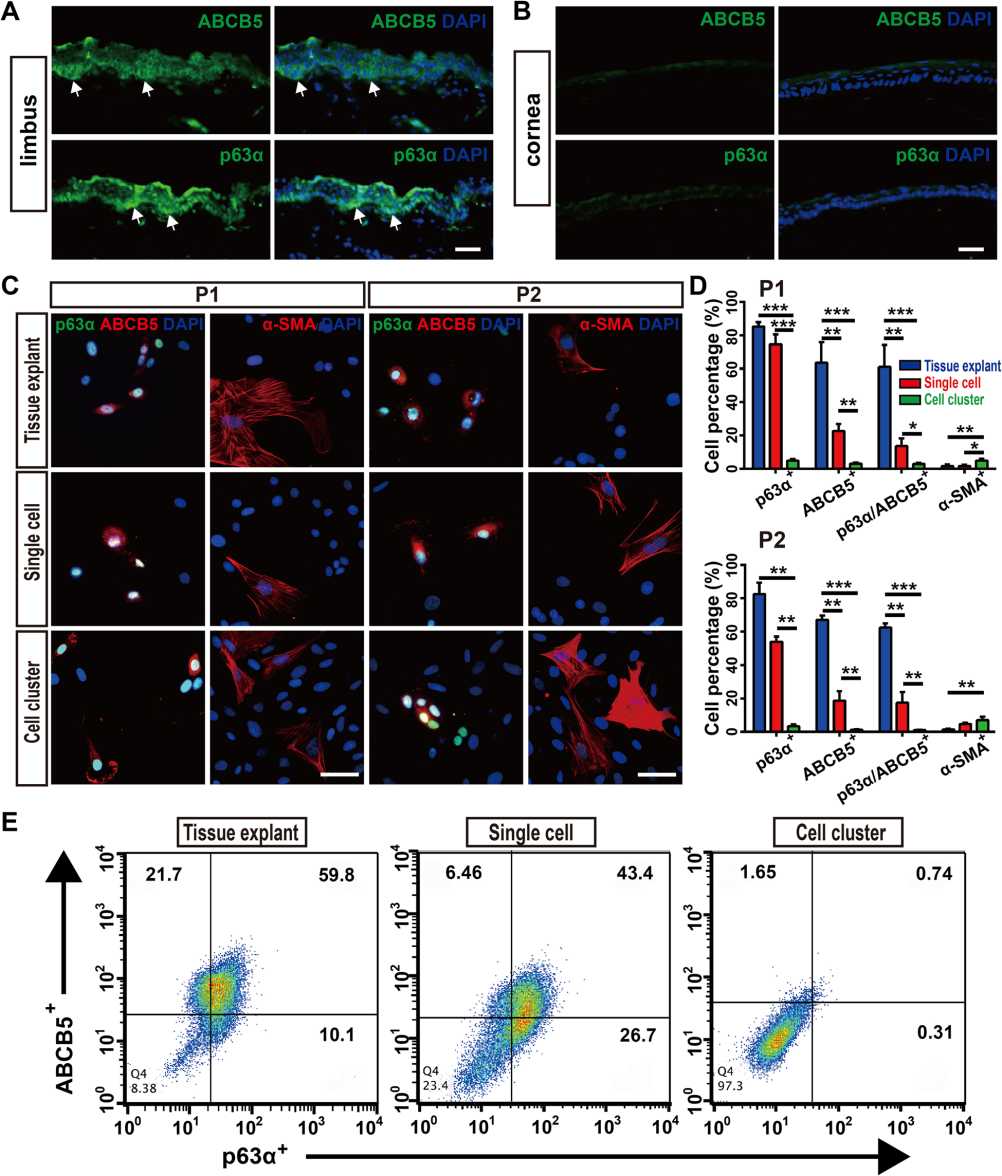
Tissue explant and single cell-suspension cultures produced more LESCs and fewer stromal cells. **a**, **b** ABCB5 and p63α positivity in rabbit limbus, with negativity in central cornea. Arrows point to the ABCB5^+^ and p63α^+^ LESCs in the limbus. **c**, **d** Expressions of proposed LESC markers (p63α and ABCB5) and stromal cell marker (α-SMA) in P1 and P2 LESCs from the three cultures. Data were given as mean ± SD from three independent experiments. One-way ANOVA analysis: *P < 0.05; **P < 0.01; ***P < 0.001. **e** Flow cytometric staining (gating based on control staining) for p63α and ABCB5 of LESCs (P1) from the three cultures. Scale bar, 50 μm. ABCB5 sub-family B, member 5, α-SMA alpha-smooth muscle actin

The phenotypes of the freshly isolated LESCs were evaluated by immunofluorescence staining for their expressions of proposed LESC markers, such as ATP-binding cassette, sub-family B, member 5 (ABCB5), and nuclear protein p63α [31, 32]. We first examined whether ABCB5 and p63α marked rabbit LESCs. We found that ABCB5^+^ cells and p63α^+^ cells were located in basal limbal epithelium of rabbit, but not center cornea (Fig. 2a, b). Then, we evaluated the expression of p63α and ABCB5 in cultured LESCs from the three cultures. The proposed LESC markers, p63α and ABCB5, were strongly expressed in LESCs from tissue explant cultures. In single cell-suspension cultures, slightly fewer cells expressed p63α and ABCB5 (Fig. 2c, d). However, cell cluster cultures expressed the lowest p63α and ABCB5, which was further confirmed by flow cytometric analysis for p63α and ABCB5 (Fig. 2c–e).

More α-SMA^+^ stromal cells were present in the cell cluster cultures than in single cell-suspension cultures, probably as a result of deeper digestion and more stromal cells obtained by the collagenase than by the dispase. In the three cultures, LESCs from tissue explant cultures expressed the lowest α-SMA (Fig. 2c, d).

### CFE and corneal epithelial differentiation of LESCs from the three cultures

**Fig. 3:**
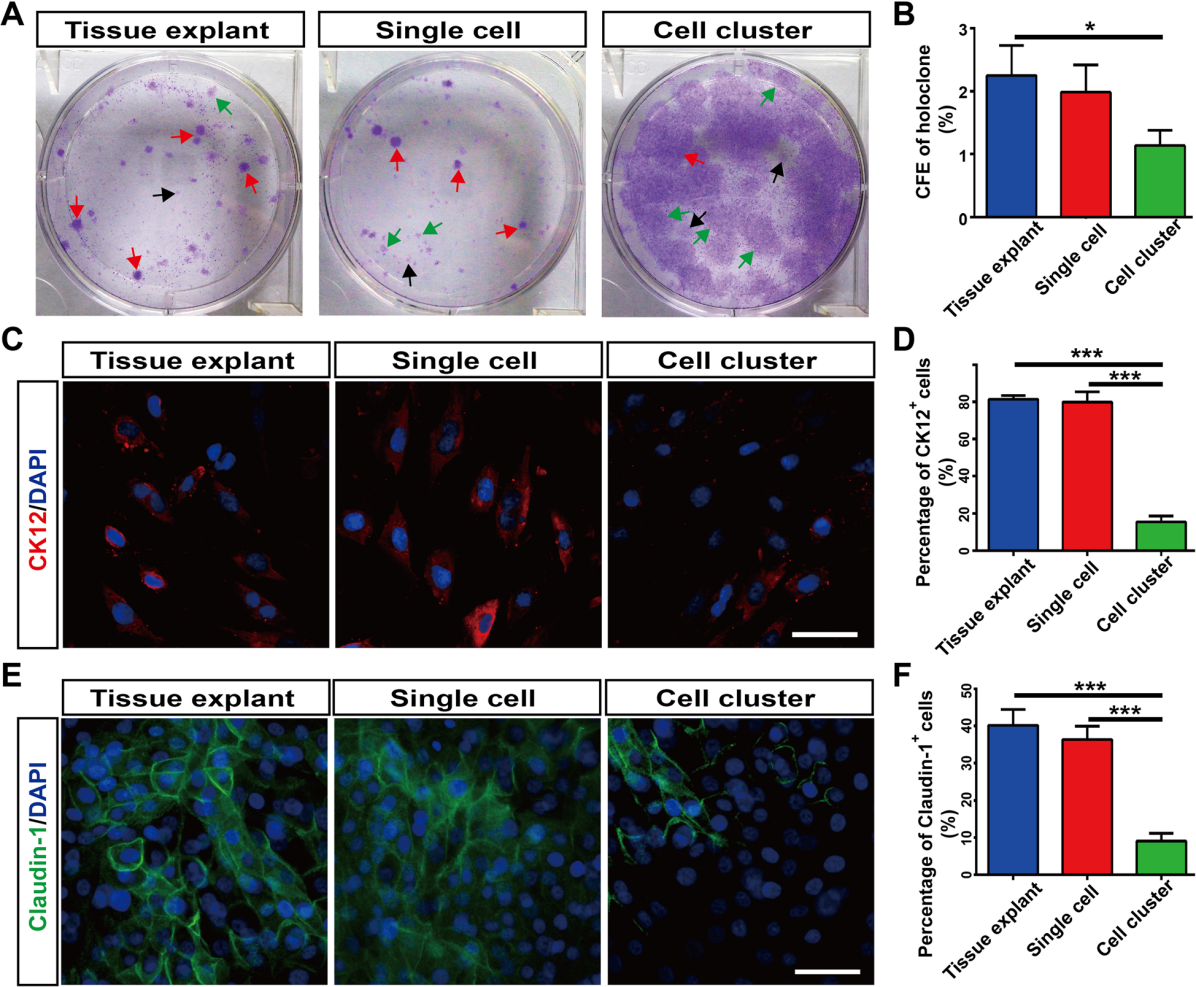
Colony forming efficiency (CFE) and corneal epithelial cell marker expression from the three cultures. **a**, **b** Crystal violet staining of LESC colonies from the three cultures. Red arrows point to holoclones. Green arrows point to meroclones. Black arrows point to paraclones. CFE of holoclones from the three cultures were quantified. **c**, **d** Corneal epithelial differentiation marker CK12 staining of the three cultures. Percentage of CK12^+^ cells were quantified. **e**, **f** Corneal epithelial tight junction protein Claudin-1 staining of the three cultures. Percentage of Claudin-1^+^ cells were quantified. Data were shown as mean ± SD from three independent experiments. One-way ANOVA analysis: *P < 0.05; ***P < 0.001. Scale bar, 50 μm. CK12 cytokeratin12, DAPI 4′,6-diamidino-2-phenylindole

We found that cell cluster cultures generated a higher CFE of meroclones and paraclones than tissue explant and single cell-suspension cultures (Additional file 1: Figure S1), so we only compared CFE of holoclones from the three cultures. We found that LESCs from tissue explant and single cell-suspension cultures generated higher CFE of holoclones than cell cluster cultures (Fig. 3a, b).

Next, we compared the expressions of corneal epithelial differentiation marker CK12 in the LESCs from the three cultures. The expression of CK12 was lowest in the LESCs from cell cluster cultures, indicating that fewer corneal epithelial cells were harvested in cell cluster cultures. The CK12 expressions in the tissue explant and single cell-suspension cultures were significantly higher than in cell cluster cultures, but the difference between tissue explant cultures and single cell-suspension cultures was not significant (Fig. 3c, d). The differentiation potential of LESCs was further confirmed by immunofluorescence staining for the expression of corneal epithelial tight junction protein Claudin-1. LESCs from tissue explant and single cell-suspension cultures generated more tight junctions than from cell cluster cultures; especially, LESCs from tissue explant cultures formed epithelia-like tight junctions (Fig. 3e, f). These data suggested that these cultured LESCs from tissue explant and single cell-suspension cultures harvested more corneal epithelial cells and formed more corneal epithelial tight junctions than from cell cluster cultures. Based on these results, we finally chose LESCs from tissue explant cultures as ‘seed cells’ for further LESC transplantations.

### Physicochemical properties of PEG-modified SF membranes

**Fig. 4:**
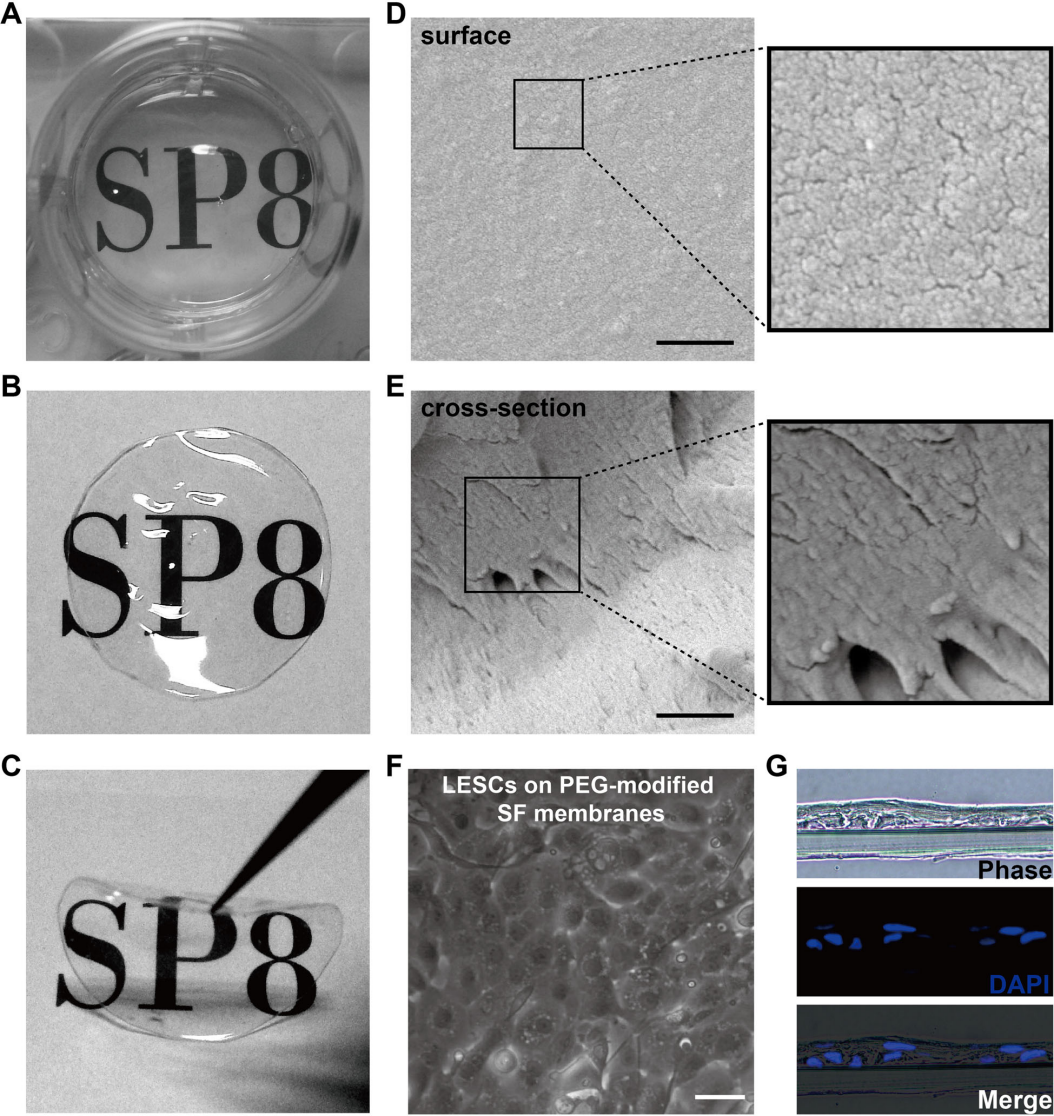
Physicochemical properties of PEG-modified SF membranes. **a** Preparation of SF membranes modified by 400-Da PEG in 12-well plates. **b**, **c** Transparent characteristics and tenacity of PEG-modified SF membranes. **d**, **e** SEM images of the surface and cross-section of PEG-modified SF membranes. **f**, **g** LESCs cultured on PEG-modified SF membranes. Scale bar, 50 μm (**f**), 200 nm (**d**, **e**). DAPI 4′,6-diamidino-2-phenylindole, LESC limbal epithelial stem cell, PEG poly(ethylene glycol), SF silk fibroin

Initially, we prepared SF membranes without PEG modification. However, the prepared SF membranes were not strong enough for surgical manipulation. A recent study reported that PEG-modified SF membranes showed significantly increased tensile strength [28]. Thus, we modified SF membranes with 400-Da PEG to enhance tensile strength, and increase the permeability and topographic roughness of SF membranes. The resulting PEG-modified SF membranes were transparent, flexible, and strong enough for surgical manipulation (Fig. 4a–c). SEM revealed that no pores were noticeable on the surface of PEG-modified SF membranes (Fig. 4d), which was consistent with a previous report [28]. The PEG-modified SF membranes showed rough morphologies of the surfaces (Fig. 4d). In cross-section, the PEG-modified SF membranes showed rough morphologies and some noticeable pores (Fig. 4e). Furthermore, LESCs cultured on PEG-modified SF membranes grew in stratified two or three layers (Fig. 4f, g).

### LESCs cultured on PEG-modified SF membranes maintain characteristics of stem cells

**Fig. 5:**
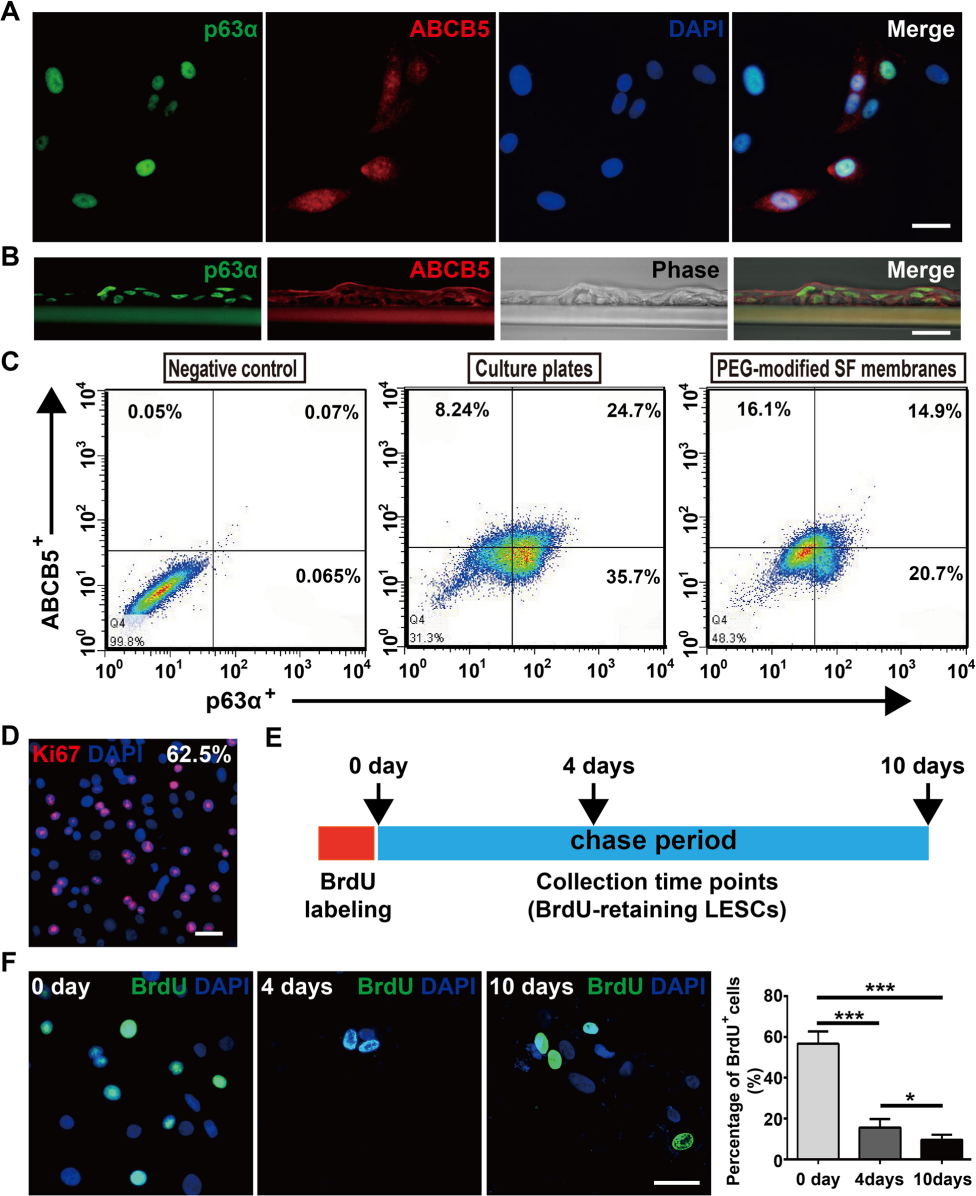
Rabbit LESCs cultured on PEG-modified SF membranes maintain characteristics of stem cells. **a**, **b** LESCs from the tissue explant cultures expressed p63α and ABCB5 on PEG-modified SF membranes. **c** Flow cytometric analysis for p63α and ABCB5 of LESCs cultured on culture plates and PEG-modified SF membranes. **d** LESCs cultured on PEG-modified SF membranes maintained high proliferative capacity (Ki67 staining). **e** Schematic summary of the experimental design for BrdU chase experiments. LESCs labeled by BrdU for 1 day, and chased in BrdU-free DMEM/F12 medium for 10 days. **f** Specific staining of BrdU-retaining LESCs cultured on PEG-modified SF membranes at 0, 4, and 10 days. Percentage of BrdU-retaining LESCs was quantified. Data was shown as mean ± SD from three independent experiments. One-way ANOVA analysis: *P < 0.05; ***P < 0.001. Scale bar, 25 μm. ABCB5 sub-family B, member 5, BrdU 5-bromo-2′-deoxyuridine, DAPI 4′,6-diamidino-2-phenylindole, LESC limbal epithelial stem cell, PEG poly(ethylene glycol), SF silk fibroin

Then, we asked whether LESCs cultured on PEG-modified SF membranes maintained characteristics of stem cells, which was critical for successful transplantations [11]. First, we examined the expression of proposed LESC markers, p63α and ABCB5, and found that LESCs cultured on PEG-modified SF membranes expressed p63α and ABCB5 (Fig. 5a, b). Flow cytometric analysis showed that, compared with cultured on six-well culture plates, the ratios of p63α^+^, ABCB5^+^, and p63α^+^/ABCB5^+^ LESCs cultured on PEG-modified SF membranes decreased. Nevertheless, the ratio of p63α^+^/ABCB5^+^ LESCs cultured on PEG-modified SF membranes was still as high as about 14.9% (Fig. 5c).

In addition, we found that LESCs cultured on PEG-modified SF membranes possessed high proliferative abilities through Ki67 staining (Fig. 5d). Then, we examined whether LESCs cultured on PEG-modified SF membranes maintained the slow-cycling characteristic of stem cells by BrdU chase experiments (Fig. 5e). After BrdU labeling for 1 day (at 0 days in the chase period), there were about 56.7% BrdU-labeling dividing LESCs, indicating high proliferative abilities of LESCs again (Fig. 5f). Furthermore, BrdU-retaining LESCs cultured on PEG-modified SF membranes decreased in the chase period (15.6% at 4 days, 9.65% at 10 days; Fig. 5f), indicating that LESCs cultured on PEG-modified SF membranes maintained the slow-cycling characteristic of stem cells and the grafts contained enough LESCs (above 3%) for successful transplantations [11].

**Fig. 6:**
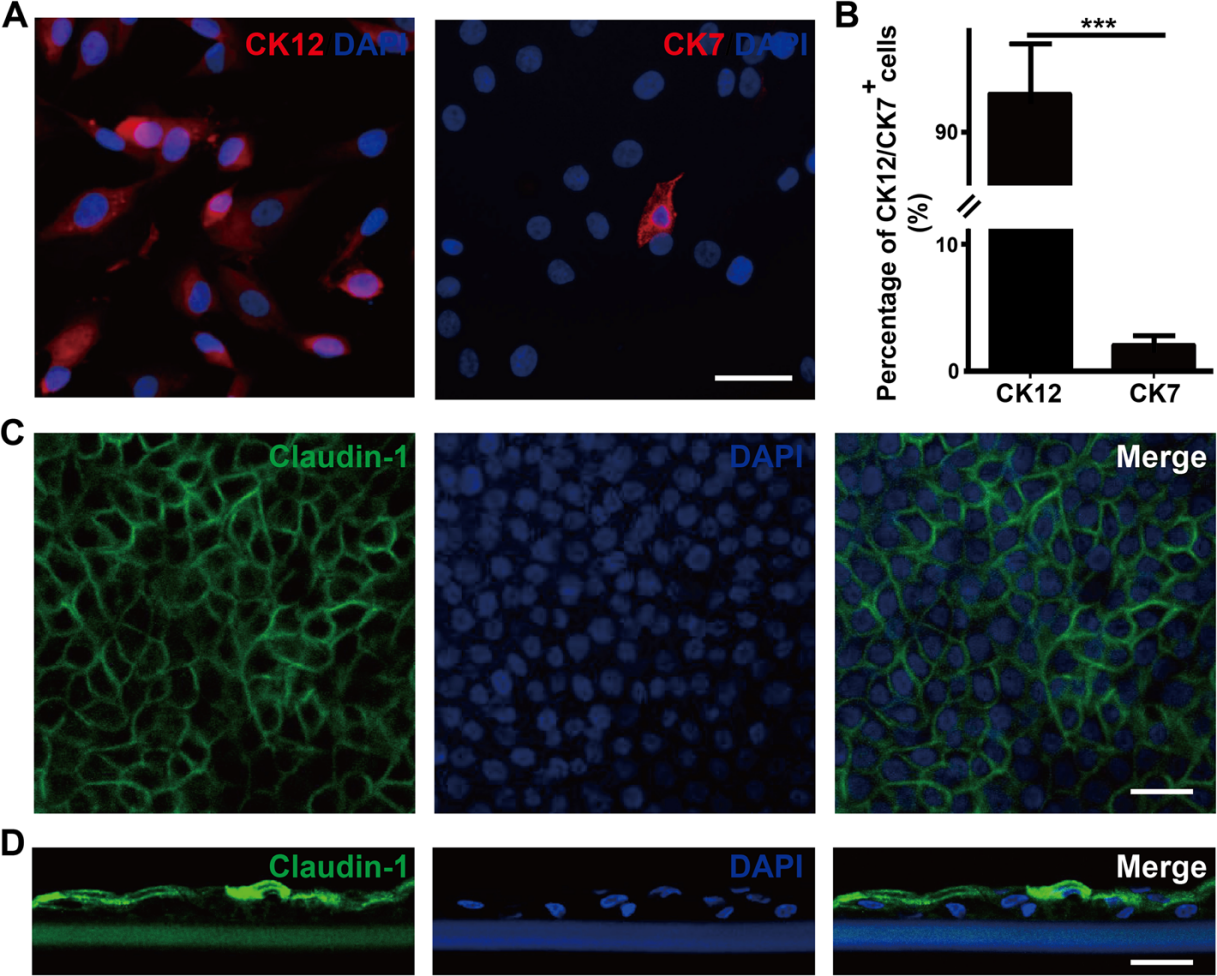
Rabbit LESCs cultured on PEG-modified SF membranes maintain normal corneal differentiation. **a**, **b** Corneal epithelial differentiation marker CK12 and conjunctival epithelial cell marker CK7 staining of LESCs cultured on PEG-modified SF membranes for 15 days. Percentages of CK12^+^ corneal epithelial cells and CK7^+^ conjunctival epithelial cells on PEG-modified SF membranes were quantified. Data was shown as mean ± SD from three independent experiments. Student’s *t* test: ***P < 0.001. **c**, **d** LESCs cultured on PEG-modified SF membranes generated corneal epithelial tight junctions (Claudin-1 staining). Scale bar, 50 μm. CK7/12 cytokeratin7/12, DAPI 4′,6-diamidino-2-phenylindole

In addition, we found that most LESCs cultured on PEG-modified SF membranes expressed corneal epithelial differentiation marker CK12, with few CK7^+^ conjunctival epithelial cells after long-time cultures (corneal epithelial cells, 94.64%; conjunctival epithelial cells, 1.85%; Fig. 6a, b). LESCs cultured on PEG-modified SF membranes also generated corneal epithelial tight junctions (Fig. 6c, d). Thus, all these data demonstrated that LESCs cultured on PEG-modified SF membranes maintained characteristics of stem cells and could differentiate into normal corneal epithelium, and PEG-modified SF membranes were suitable to be a carrier for LESC transplantation.

### Rabbit LSCD model

**Fig. 7:**
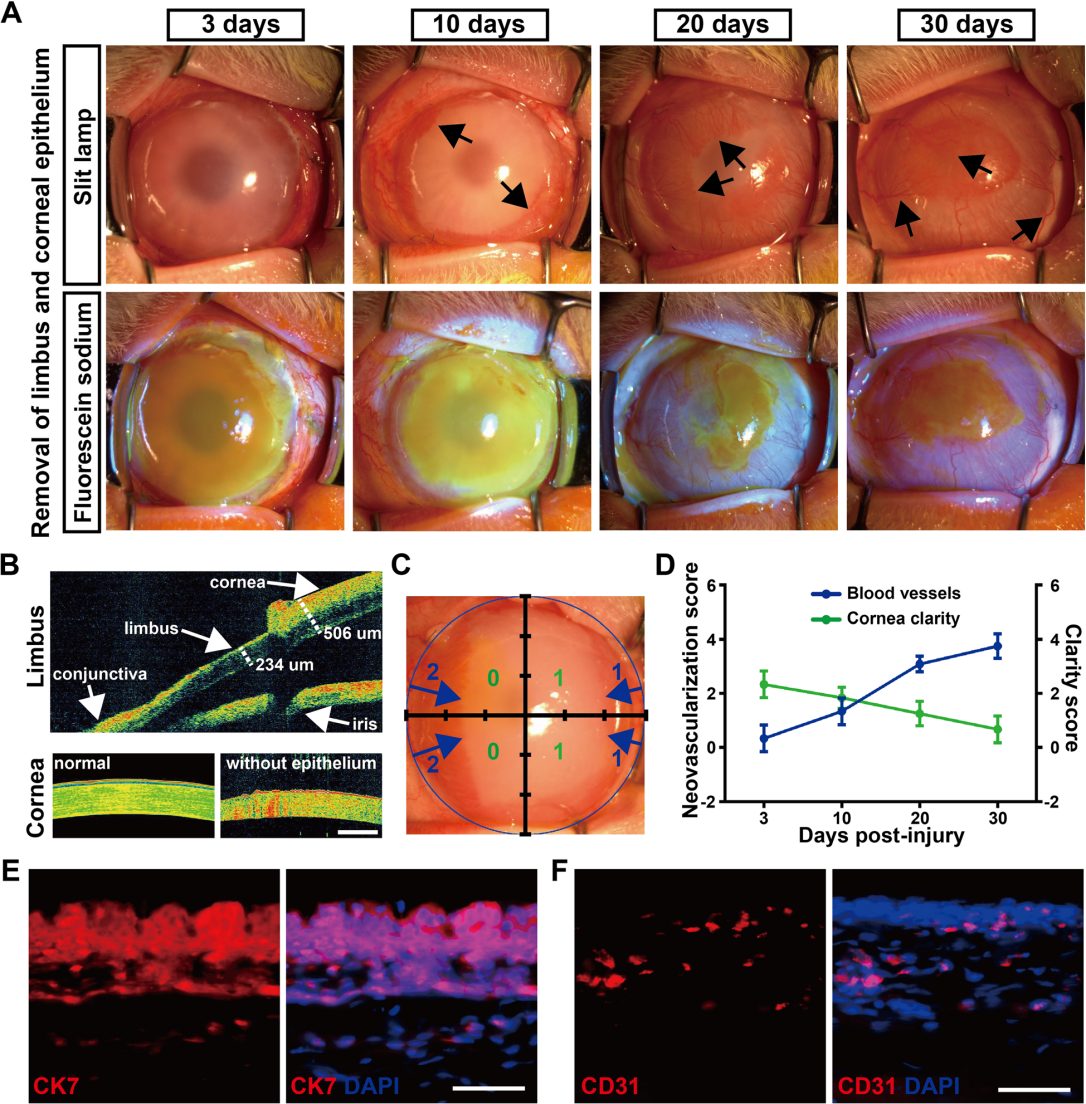
Construction of rabbit LSCD model. **a** Rabbit total LSCD model with removal of the limbus and cornea epithelium. Advanced neovascularization and epithelial defects (fluorescein sodium staining) present on the cornea. Arrows point to the advanced neovascularization and new blood vessels. **b** OCT of rabbit total LSCD model. Limbal epithelium about 270 μm in depth was removed. **c** Examples of corneal neovascularization scores (blue numbers) and clarity scores (green numbers). **d** Averaged neovascularization and corneal clarity scores at 3, 10, 20, and 30 days after surgery in rabbit total LSCD model. Data was shown as mean ± SD from three rabbits. **e**, **f** Corneas of total LSCD model exhibited LSCD-characteristic epithelial conjunctivalization (conjunctival epithelial cells marker CK7 staining) and new blood vessels (vascular endothelial cells marker CD31 staining). Scale bar, 500 μm (**b**), 50 μm (**e**, **f**). CK7 cytokeratin7, DAPI 4′,6-diamidino-2-phenylindole

The rabbit LSCD model was established by removing the limbus, with the corneal epithelium scraped off or not. We found that limbus-deficient cornea (only the limbus was removed, some termed limbal sectoral deficiency) remained transparent, and neither blood vessels nor corneal epithelial defects were observed for at least 3 months (Additional file 2: Figure S2), indicating short-term self-maintenance potential of the corneal epithelium. In the total LSCD model (removal of both limbus and corneal epithelium), new blood vessels sprouted from the limbal area in all four quadrants and began to spread toward the center cornea. After 20 days, blood vessels spread radially toward the center cornea. After 30 days, progressive vascularization covered the entire cornea, and epithelial defects verified by fluorescein sodium staining remained at the center cornea (Fig. 7a). The fluorescein sodium staining pattern denoted a poor epithelial barrier caused by conjunctivalization, a hallmark of LSCD. Optical coherence tomography (OCT) showed the removal of the limbus (about 270 μm in depth, deep enough to remove all LESCs) and corneal epithelium in the total LSCD model (Fig. 7b), and proposed LESC marker (p63α and ABCB5) staining confirmed the LESC deficiency in the limbus (Additional file 2: Figure S2C). Scores of corneal neovascularization and clarity were shown at all evaluation times, indicating a successful LSCD model with increased neovascularization and decreased corneal clarity (Fig. 7c, d). Furthermore, the total LSCD model exhibited LSCD-characteristic epithelial conjunctivalization (conjunctival epithelial cells marker CK7 staining) and new blood vessels (vascular endothelial cell marker CD31 staining) in the cornea (Fig. 7e, f). Taken together, we constructed a rabbit total LSCD model successfully for further LESC transplantations.

### Restoration of LSCD after LESC/SF graft transplantation

**Fig. 8:**
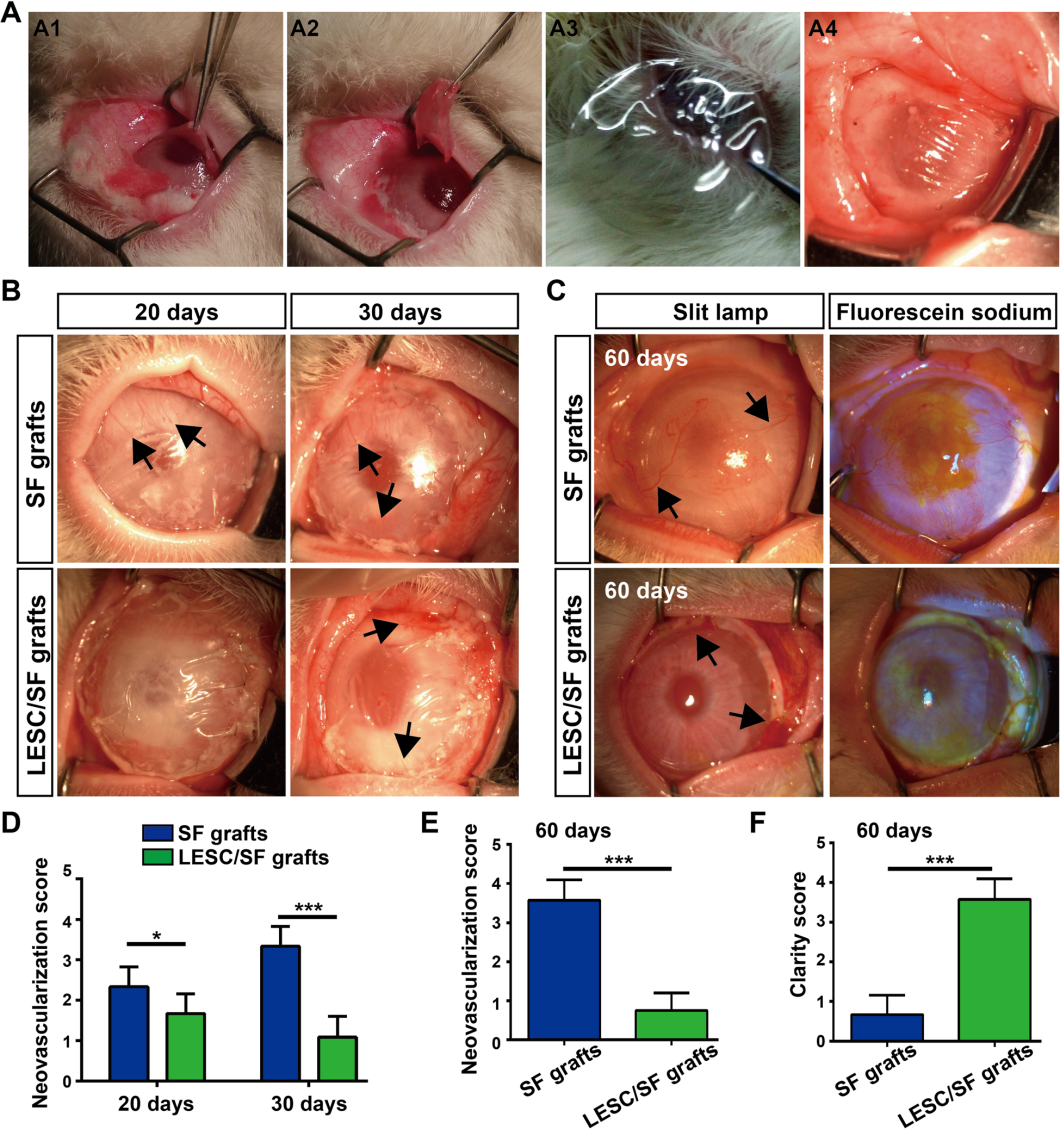
Restoration of LSCD by LESC/SF graft transplantation. **a** Surgical procedure of LESC/SF graft transplantation. Corneal vascular pannus were removed (**A1**, **A2**), and PEG-modified SF membranes with or without LESCs were secured with interrupted 10–0 nylon sutures just beyond the limbus (**A3**, **A4**). **b** PEG-modified SF membranes (SF grafts) and LESCs cultured on PEG-modified SF membranes (LESC/SF grafts) were transplanted onto the corneas with LSCD. Representative images of rabbit corneas at 20 and 30 days after transplantation. **c** Neovascularization and epithelial defects images of rabbit corneas at 60 days after transplantation. Arrows point to the new blood vessels. **d**–**f** Neovascularization and clarity scores of rabbit corneas were quantified at 20, 30, and 60 days after transplantation. Data was shown as mean ± SD from three rabbits. Student’s *t* test: *P < 0.05; ***P < 0.001. LESC limbal epithelial stem cell, SF silk fibroin

Then, we chose the rabbit total LSCD model to transplant LESCs cultured on PEG-modified SF membranes (LESC/SF grafts). After removal of the fibrovascular pannus, PEG-modified SF membranes (SF grafts) or LESC/SF grafts were transplanted onto rabbit corneas with LSCD (Fig. 8a). In the SF grafts group, all eyes remained inflamed and new blood vessels spread toward the center cornea at 20 and 30 days (Fig. 8b). At 60 days, corneas were completely vascularized and epithelial defects were verified by fluorescein sodium staining (Fig. 8c). In the LESC/SF grafts group, all eyes remained inflamed and inflammation reduced gradually at 20 and 30 days. At 30 days, a few new blood vessels appeared in the limbus, and these new blood vessels vanished gradually (Fig. 8b). At 60 days, after removal of PEG-modified SF membranes, only few blood vessels existed in the limbus and few diffuse corneal epithelial defects were observed on the cornea (Fig. 8c). At all evaluation times, scores of corneal neovascularization in the LESC/SF grafts group were significantly lower than those in the SF grafts group (Fig. 8d, e). Furthermore, corneal clarity in the LESC/SF grafts group was better than in the SF grafts group (Fig. 8f). These LESC/SF graft transplantations were rated success as the corneas became clear and smooth.

### Corneal epithelial regeneration and repair after LESC/SF graft transplantation

**Fig. 9:**
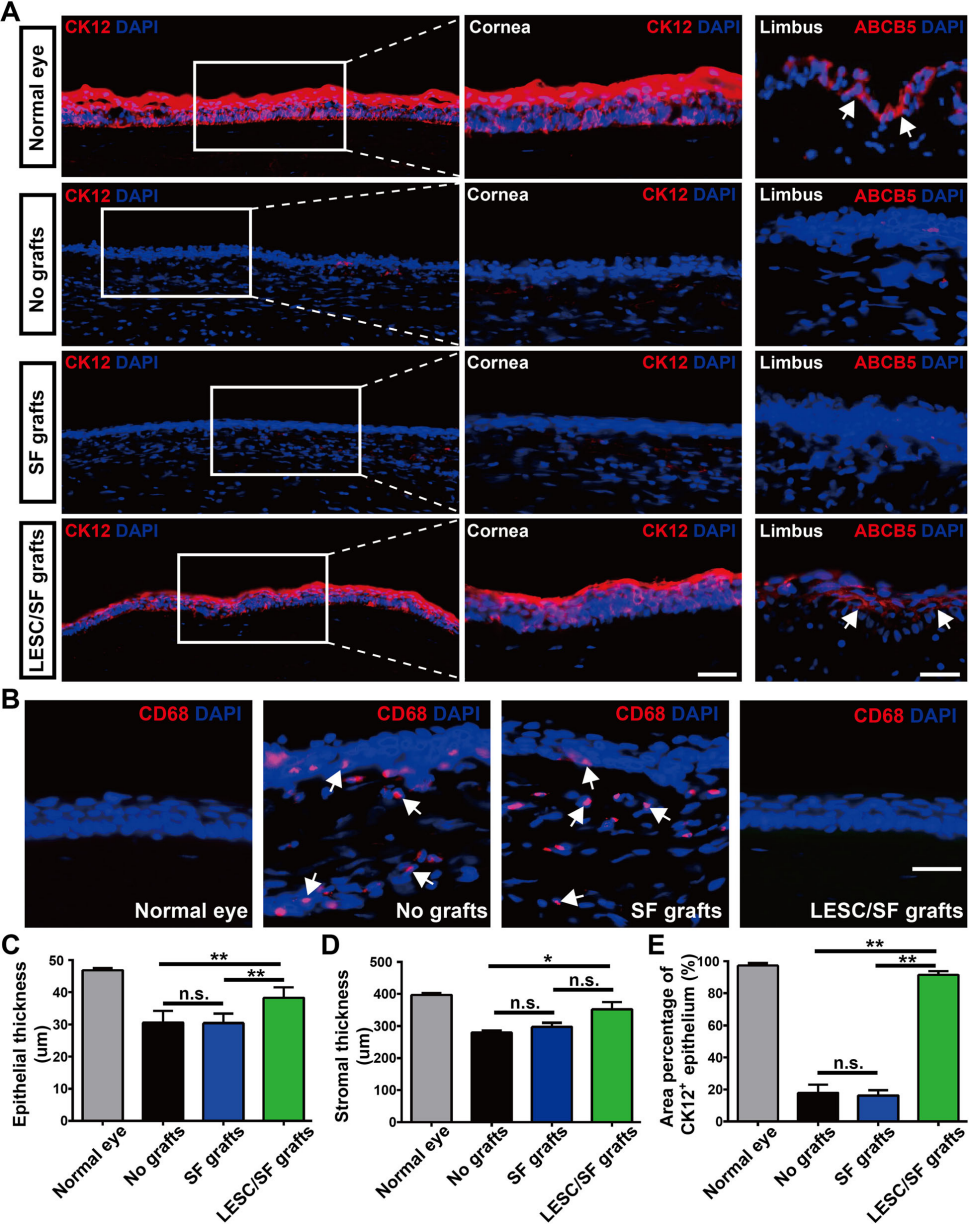
Corneal epithelial regeneration and repair after LESC/SF graft transplantation. **a** Rabbit corneas 4 months after transplantation (left panels, corneal epithelial cells marker CK12 staining; middle panels, enlarged pictures of the framed area; right panels, proposed LESC marker ABCB5 staining in the limbus). The four groups are as follows: (1) cornea from normal eyes (positive control), (2) cornea from LSCD model (no grafts, negative control), (3) PEG-modified SF membrane transplantation (SF grafts), and (4) LESCs cultured on PEG-modified SF membrane transplantation (LESC/SF grafts). Arrows point to the ABCB5^+^ LESCs in the limbus. **b** Macrophage antigen CD68 (located at endosome, lysosome, and cell membrane) staining showed inhibited inflammation in the corneas 4 months after LESC/SF graft transplantations. Arrows point to CD68^+^ macrophages in the corneas. **c** Corneal epithelial thickness in the four groups. **d** Corneal stromal thickness in the four groups. **e** Area percentages of CK12^+^ epithelium in total corneal epithelium in the four groups. Transplantation experiments were performed in three rabbits per group. Three consecutive cross-sections from three rabbits per group were analyzed. Data was shown as mean ± SD. One-way ANOVA: *P < 0.05; **P < 0.01; n.s. not significant. Scale bar, 50 μm. ABCB5 sub-family B, member 5, CK12 cytokeratin12, DAPI 4′,6-diamidino-2-phenylindole, LESC limbal epithelial stem cell, SF silk fibroin

To further confirm the restoration of LSCD by LESC/SF grafts, we evaluated the corneal epithelial regeneration and repair 4 months after transplantation. We showed that normal corneas had a stratified epithelial layer with positive staining of corneal-specific CK12, and corneas from the LESC/SF grafts group formed a continuous sheet of CK12^+^ corneal epithelial cells with inhibited inflammation 4 months after transplantation (Fig. 9a, b and Additional file 3: Figure S3). In contrast, corneas from the no grafts (LSCD model) and SF grafts groups displayed subepithelial scarring, epithelial conjunctivalization, corneal neovascularization, and inflammation (Fig. 9a, b and Additional file 3: Figure S3). We found that more transplanted LESCs survived in the limbal region but not in the cornea, and these surviving LESCs repopulated the limbus as evidenced by a large number of ABCB5^+^ LESCs in the limbus (Additional file 3: Figure S3A, C). Furthermore, LESC/SF grafts increased corneal epithelial thickness, stromal thickness, and the area percentage of CK12^+^ corneal epithelium (Fig. 9c–e). Notably, these surviving LESCs in the limbus were capable of repairing large corneal epithelial defects once again after corneal epithelial scraping (Additional file 3: Figure S3D). Taken together, these data demonstrated that LESCs cultured on PEG-modified SF membranes were able to repair corneal surface defects and reverse LSCD, and PEG-modified SF membranes were suitable to be a carrier for LESC transplantation.

## Discussion

To maintain ocular surface stability and optical transparency and protect against the external environment, corneal epithelia are constantly renewed, which is governed by LESCs. Although LESCs are known to reside mainly in the palisades of Vogt, several studies suggest that they may lie deeper than the basement membrane plane. With the advances in tissue engineering techniques, cultured LESC transplantation has been a better choice to treat LSCD. Many factors, including the methods of isolation and the substrates or carriers that support LESCs, can influence the quality of the in-vitro expanded LESCs and the outcomes following transplantation. In this study, we comprehensively compared three culture methods of LESCs in parallel to determine the most applicable expansion method of LESCs in vitro for ocular surface reconstruction. In addition, we prepared SF membranes modified by 400-Da PEG to culture LESCs and transplanted these LESC/SF grafts onto a rabbit LSCD model. Our results suggested that tissue explant and single cell-suspension cultures of LESCs were superior to cell cluster cultures for direct application of LESC transplantation, and PEG-modified SF membranes were suitable to be a carrier for LESC transplantation.

Because of an array of desirable properties, including low immune response, suitable mechanical strength, and permeable for oxygen, fluids, and biomolecules, SF membranes have been investigated extensively as biomaterials for corneal epithelial cell growth and ocular surface reconstruction. The presence of pores in the SF membranes increases permeability and diffusion of oxygen, nutrients, and biomolecules that must be supplied to the cells to regenerate tissue, which is beneficial for the cells’ growth on the SF membranes. In addition, porosity also has favorable effects on the intercellular communication and signaling where the cells are expected to operate. On the nonporous membranes, cultivation of human corneal limbal epithelial cells (HCLECs) for 2 weeks resulted in stratified layers of cells with a basal cuboidal layer. In contrast, cells on the porous membranes modified by 900-kDa PEG formed a flattened and squamous monolayer. It seems that porous membranes induced by PEG of high MW appear to offer no advantage for cell growth [28]. In the present study, we modified SF membranes with 400-Da PEG, with an expectation of roughness and generating topographic features on the surface of the SF membranes. Similar to the growth on the nonporous membranes, LESCs cultured on the SF membranes modified by 400-kDa PEG grew in stratified two or three layers. The reason for this may be that larger pores in the SF membranes create a surface topography that is less attractive to the LESCs and therefore reduce growth and stratification of the cells. Furthermore, we showed that LESCs cultured on the PEG-modified SF membranes maintained the characteristics of stem cells and differentiated into normal corneal epithelium. Combined with other reports, it appears that the precise mechanism of PEG action is not only involved in the size of pores, but further aspects like topographic features created by PEG on the surface may also affect the outcomes of LESC cultures.

The percentage of stem cells in cultured LESCs is associated with clinical success, so carriers used for LESC cultivation and transplantation must maintain characteristics of stem cells and support the growth and differentiation of LESCs. Although LESCs cultured on the PEG-modified SF membranes maintained characteristics of LESCs, we also found a decrease of p63α^+^/ABCB5^+^ LESCs. Increasing evidence supports that somatic stem cells are regulated by their niches, a special microenvironment consisting of other cellular and extracellular components in the vicinity [33, 34]. Some studies demonstrated that the limbal stromal microenvironment indeed played an important role in downregulating epithelial differentiation, and that limbal stroma might contain niche cells to maintain the LESC phenotype [35, 36]. These niche cells provided a sheltering environment that shielded LESCs from stimuli that adversely promoted differentiation and apoptosis [34]. Earlier observations reported that the function of LESCs depended on close physical cell–cell contact with their native stromal niche cells (PCK^–^/Vim^+^) via SDF-1/CXCR4 signaling to maintain LESCs in an undifferentiated state during in-vitro expansion [15, 16]. Another report showed that the limbal stroma modulated epithelial differentiation, proliferation, and apoptosis in the direction favoring stemness, whereas the corneal stroma promoted differentiation [35]. Thus, the influence of the stromal microenvironment is imperative to recreate the stem cell niche and promote optimal LESC function and long-term limbal graft success. Higa et al. [37] reported that a 3 T3 feeder layer was useful for LESCs cultured on SF membranes to develop a normal corneal phenotype and retain good progenitor cell activity. Bray et al. [24] reported that dual-layer fibroin scaffolds consisting of LESCs and limbal mesenchymal stromal cells (LMSCs) maintained a similar phenotype to that on the separate layers. However, although the presence of LMSCs is beneficial for in-vitro expansion of LESCs, it may not be necessary to transplant them in large numbers for future therapeutic delivery to restore the stem cell niche following transplantation.

Although LESCs cultured on the PEG-modified SF membranes maintain characteristics of stem cells and differentiate into normal corneal epithelium, SF membranes present their own challenges like many other biomaterials of natural origin. The amino acid sequence of SF favors the formation of β-sheet structures which are exceptionally stable and hydrophobic in nature. As a result, SF-based materials degrade more slowly than other commonly used materials including collagen, fibrin, and polylactides. A faster rate of degradation may be helpful in avoiding chronic foreign body reactions. Thus, further studies should give high priority to biodegradation and biocompatibility of SF membranes. Composite biomaterials may be a feasible solution. Some biomaterial such as polycaprolactone (PCL), a biodegradable aliphatic polyester with high tensile and elongation properties approved by the US Food and Drug Administration (FDA) for the human body—may promote degradation and improve biocompatibility of SF membranes.

The Holy Grail of LESC research is to find a distinct phenotype or specific cell marker that allows stem cells to be distinguished from all neighboring cells, including early transient amplifying cells (eTACs), and to be isolated for further studies. Many candidate markers, including p63α [32], ABCG2 [38], and IPO13 [39], have been proposed based on differential expression studies or conventional immunostaining. Ksander et al. [31] reported that ABCB5 might be used as a cell marker of LESCs and was required in the LESC maintenance, corneal development and regeneration in murine and human. More importantly, isolated enriched human ABCB5^+^ limbal cells are exclusively capable of reversing LSCD through long-term corneal regeneration. This work promotes further advances in the clinical applications of cultured LESCs for ocular surface reconstruction. However, our results showed stromal staining of ABCB5 in the limbus, and some LESCs were ABCB5^+^/p63α^–^. A recent study reported that human limbal mesenchymal stem cells also expressed ABCB5 [40]. In addition, previous studies found that ABCB5 was expressed in skin progenitor cells and melanoma stem cells, and functioned as a regulator of cellular differentiation [41, 42]. These data raise questions: whether ABCB5 only marks LESCs in the limbus and whether ABCB5 is a definitive marker of LESCs. Interestingly, two widely proposed LESC markers, p63α and ABCG2, were also expressed in human limbal mesenchymal stem cells [40], and ABCG2 was also expressed in Langerhans cells rather than LESCs in the limbus [43]. Thus, we believe that all of these proposed LESC markers are not definitive cell markers only for LESCs. Nevertheless, these proposed LESC markers (e.g., ∆Np63α) have been proven useful for ensuring sufficient LESCs and eTACs for successful clinical transplantations [11]. Furthermore, because of its expression on cellular membrane, ABCB5 will be a useful cell marker for sorting LESCs and/or eTACs for further studies and applications. In addition, a study showed that stromal stem cells supported limbal epithelial cells on Real Architecture For 3D Tissue (RAFT) tissue equivalents [44], and whether these mesenchymal stem cells or stromal stem cells are helpful for clinical transplantation requires further study.

The corneal epithelium is a squamous epithelium that is constantly renewing, with a vertical turnover of 1–2 weeks in many mammals. However, rabbit central corneal epithelium can remain apparently intact for a long time in the limbus-deficient model, indicating that central cornea epithelium has a significant self-maintenance potential. There are reports of cornea remaining transparent for years in limbal deficiency [45]. This raises the question of whether there exist corneal epithelial progenitor cells in the central cornea. Majo et al. [46] reported that the limbus was not the only niche for corneal stem cells and oligopotent stem cells were distributed throughout the mammalian ocular surface. This study leads to the corneal epithelial stem cell (CESC) hypothesis, which postulates that stem cells distribute throughout the basal corneal epithelium and maintain the corneal epithelium during normal homeostasis. According to this hypothesis, LESCs are present in the limbus but are only activated during wound healing. However, recent lineage tracing studies supported the LESC hypothesis which proposed that LESCs in the limbus maintained the corneal epithelium both during normal homeostasis and wound repair [47–49]. Interestingly, corneal differentiation was found in human conjunctiva, and conjunctival cells could be transplanted successfully in the human to replace cornea [30, 50]. How does the central corneal epithelium remain apparently intact in the limbus-deficient cornea? What signaling pathway regulates the self-maintenance potential of cornea after the removal of limbus? Answers to these questions will provide new insight into future treatments for LSCD.

## Conclusions

We confirmed the feasibility of using PEG-modified SF membrane as a carrier for long-term sustainable culture and growth of functional LESCs. We found that LESCs cultured on PEG-modified SF membranes maintained characteristics of stem cells and normal corneal differentiation. After transplantation onto a rabbit LSCD model, LESC/SF grafts inhibited new blood vessels, rescued corneal epithelial defects, and repopulated the limbus and the corneas became clear and smooth. Therefore, PEG-modified SF membranes are appropriate candidates as a substitute for donor AM as a carrier for LESC transplantation. Long-term observations are required before this carrier can be adopted for clinical treatment.

## Additional files

Additional file 1: Is Figure S1 showing CFE of LESCs from the three cultures. (**A**) Definitions of holoclone, meroclone, and paraclone. Holoclones defined as large round colonies with smooth and regular borders and formed entirely by small epithelial cells. Meroclones defined as large colonies formed by small epithelial cells but showing irregular borders and/or with areas containing large stromal cells. Paraclones defined as small colonies with wrinkled and irregular borders and formed by large cells. Arrows point to small epithelial cells. Arrowheads point to large stromal cells. Scale bar, 50 μm. (**B**) CFE of holoclones, meroclones, and paraclones, and total CFE. (**C**) Percentages of CFE of holoclones, meroclones, and paraclones in total CFE of LESCs from the three cultures. Data was shown as mean ± SD from three experiments. One-way ANOVA: *P < 0.05; **P < 0.01. (JPG 2106 kb)

Additional file 2: Is Figure S2 showing rabbit limbus-deficient model with removal of limbus only. (**A**) Corneas of rabbit limbus-deficient model (some termed limbal sectorial deficiency) remained transparent for at least 3 months. Neovascularization and epithelial defects (fluorescein sodium staining) not present on the cornea. (**B**) Corneal neovascularization scores and clarity scores of the limbus-deficient model at 10, 30, 60, and 90 days after the removal of limbus. Data was shown as mean ± SD from three rabbits. (**C**) Proposed LESC marker (p63α and ABCB5) staining of the limbus-deficient model in the limbus showed LESC deficiency following removal of limbus. (**D**) Rabbit corneas of limbus-deficient model did not exhibit LSCD-characteristic epithelial conjunctivalization (CK7 staining) and new blood vessels (vascular endothelial cells marker CD31 staining), indicating short-term self-maintenance potential of the corneal epithelium. Scale bar, 50 μm. (JPG 3985 kb)

Additional file 3: Is Figure S3 showing restoration of LSCD and repopulated limbus by LESC/SF graft transplantation. (**A**) Rabbit corneas 2 months after LESC/SF graft transplantation (left panel, corneal epithelial cells marker CK12 staining; middle panel, enlarged pictures of the framed area; right panel, proposed LESCs marker ABCB5 staining in the limbus). Before LESC/SF graft transplantation, LESCs were labeled by DiO (DiO-LESCs, green) to trace these donor LESCs. More transplanted LESCs survived in the limbal region, but not in the cornea. Arrows point to ABCB5^+^ LESCs in the limbus. (**B**) Rabbit corneas 4 months after transplantation (left panels, HE staining; middle panels, enlarged pictures of the framed area; right panels, conjunctival epithelial cells marker CK7 staining and vascular endothelial cells marker CD31 staining). Normal corneas showed typical corneal epithelium. Corneas from no grafts (LSCD model) and SF grafts groups showed epithelial conjunctivalization and new blood vessels. Corneas from LESC/SF grafts group showed healed cornea surface without conjunctival epithelial cells and blood vessels. (**C**) LESC restoration in the limbus by LESC/SF grafts. ABCB5^+^ LESCs only existed in the limbal region but not in the cornea 4 months after LESC/SF transplantation, indicating that stem cell niche in the limbus was favorable for transplanted LESC survival and growth. (**D**) Repair of injured corneal epithelium once again. Top panels, regenerated corneal epithelium 4 months after initial LESC/SF graft transplantations was scraped off and made a large corneal epithelium defect (arrows). Bottom panels, injured corneal epithelium restored once again within 3 days with healed epithelial defect. Scale bar, 50 μm. (JPG 7374 kb)

## Abbreviations

ABCB5: ATP-binding cassette, sub-family B, member 5
ABCG2: ATP-binding cassette, sub-family G, member 2
AL: Air-lifting
AM: Amniotic membrane
BrdU: 5-Bromo-2′-deoxyuridine
BSA: Bovine serum albumin
CESC: Corneal epithelial stem cell
CFE: Colony forming efficiency
CK12: Cytokeratin 12
CK7: Cytokeratin 7
DAPI: 4′, 6-Diamidino-2-phenylindole
ECM: Extracellular matrix
eTAC: Early transient amplifying cell
FBS: Fetal bovine serum
HCLEC: Human corneal limbal epithelial cell
HE: Hematoxylin and eosin
IgG: Immunoglobulin G
IPO13: Importin 13
KSFM: Keratinocyte serum-free medium
LESC: Limbal epithelial stem cell
LMSC: Limbal mesenchymal stromal cell
LSCD: Limbal stem cell deficiency
MW: Molecular weight
OCT: Optical coherence tomography
PCL: Polycaprolactone
PEG: Poly(ethylene glycol)
PFA: Paraformaldehyde
RAFT: Real architecture for 3D tissue
SD: Standard deviation
SEM: Scanning electron microscopy
SF: Silk fibroin
α-SMA: Alpha smooth muscle actin
