# Supplementary figures and images for: Poly(ethylene glycol)-modified silk fibroin membrane as a carrier for limbal epithelial stem cell transplantation in a rabbit LSCD model
Source: Stem Cell Res Ther. 2017 Nov 7;8:256. doi: 10.1186/s13287-017-0707-y (PMC5678789; doi:10.1186/s13287-017-0707-y)

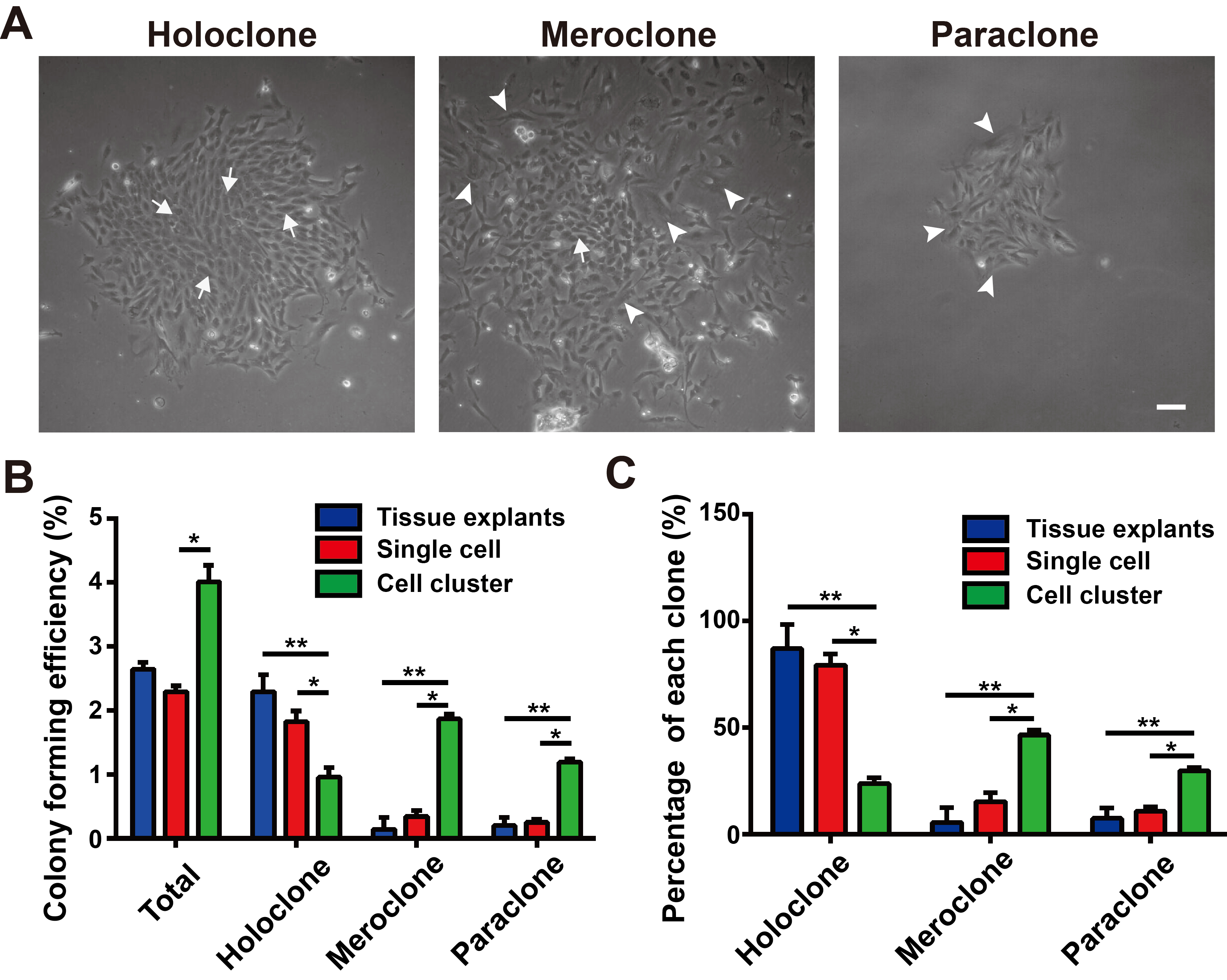

Supplement: Supplementary file 1 — Is Figure S1 showing CFE of LESCs from the three cultures. (A) Definitions of holoclone, meroclone, and paraclone. Holoclones defined as large round colonies with smooth and regular borders and formed entirely by small epithelial cells. Meroclones defined as large colonies formed by small epithelial cells but showing irregular borders and/or with areas containing large stromal cells. Paraclones defined as small colonies with wrinkled and irregular borders and formed by large cells. Arrows point to small epithelial cells. Arrowheads point to large stromal cells. Scale bar, 50 μm. (B) CFE of holoclones, meroclones, and paraclones, and total CFE. (C) Percentages of CFE of holoclones, meroclones, and paraclones in total CFE of LESCs from the three cultures. Data was shown as mean ± SD from three experiments. One-way ANOVA: *P < 0.05; **P < 0.01. (JPG 2106 kb) [file 13287_2017_707_MOESM1_ESM.jpg]

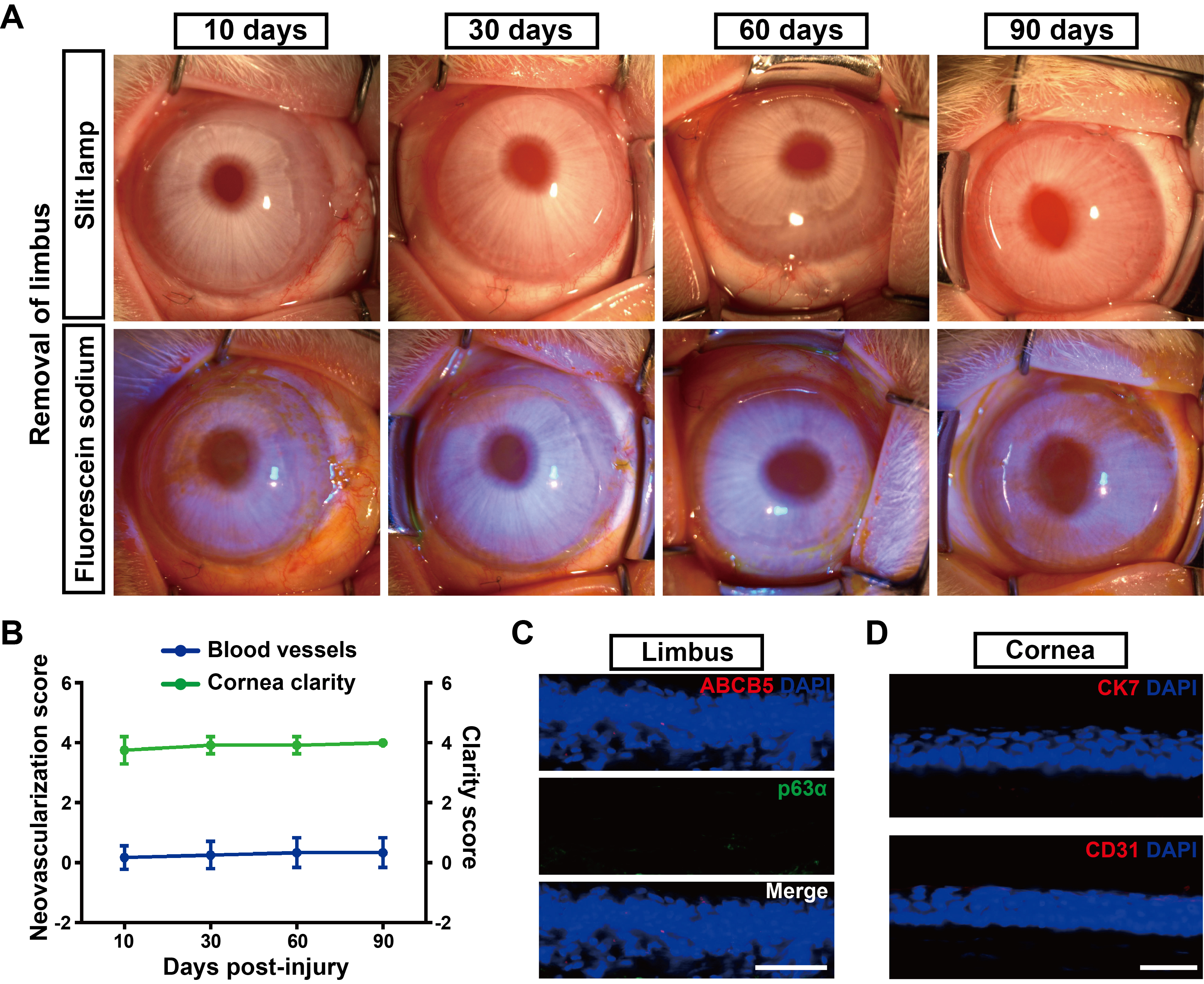

Supplement: Supplementary file 2 — Is Figure S2 showing rabbit limbus-deficient model with removal of limbus only. (A) Corneas of rabbit limbus-deficient model (some termed limbal sectorial deficiency) remained transparent for at least 3 months. Neovascularization and epithelial defects (fluorescein sodium staining) not present on the cornea. (B) Corneal neovascularization scores and clarity scores of the limbus-deficient model at 10, 30, 60, and 90 days after the removal of limbus. Data was shown as mean ± SD from three rabbits. (C) Proposed LESC marker (p63α and ABCB5) staining of the limbus-deficient model in the limbus showed LESC deficiency following removal of limbus. (D) Rabbit corneas of limbus-deficient model did not exhibit LSCD-characteristic epithelial conjunctivalization (CK7 staining) and new blood vessels (vascular endothelial cells marker CD31 staining), indicating short-term self-maintenance potential of the corneal epithelium. Scale bar, 50 μm. (JPG 3985 kb) [file 13287_2017_707_MOESM2_ESM.jpg]

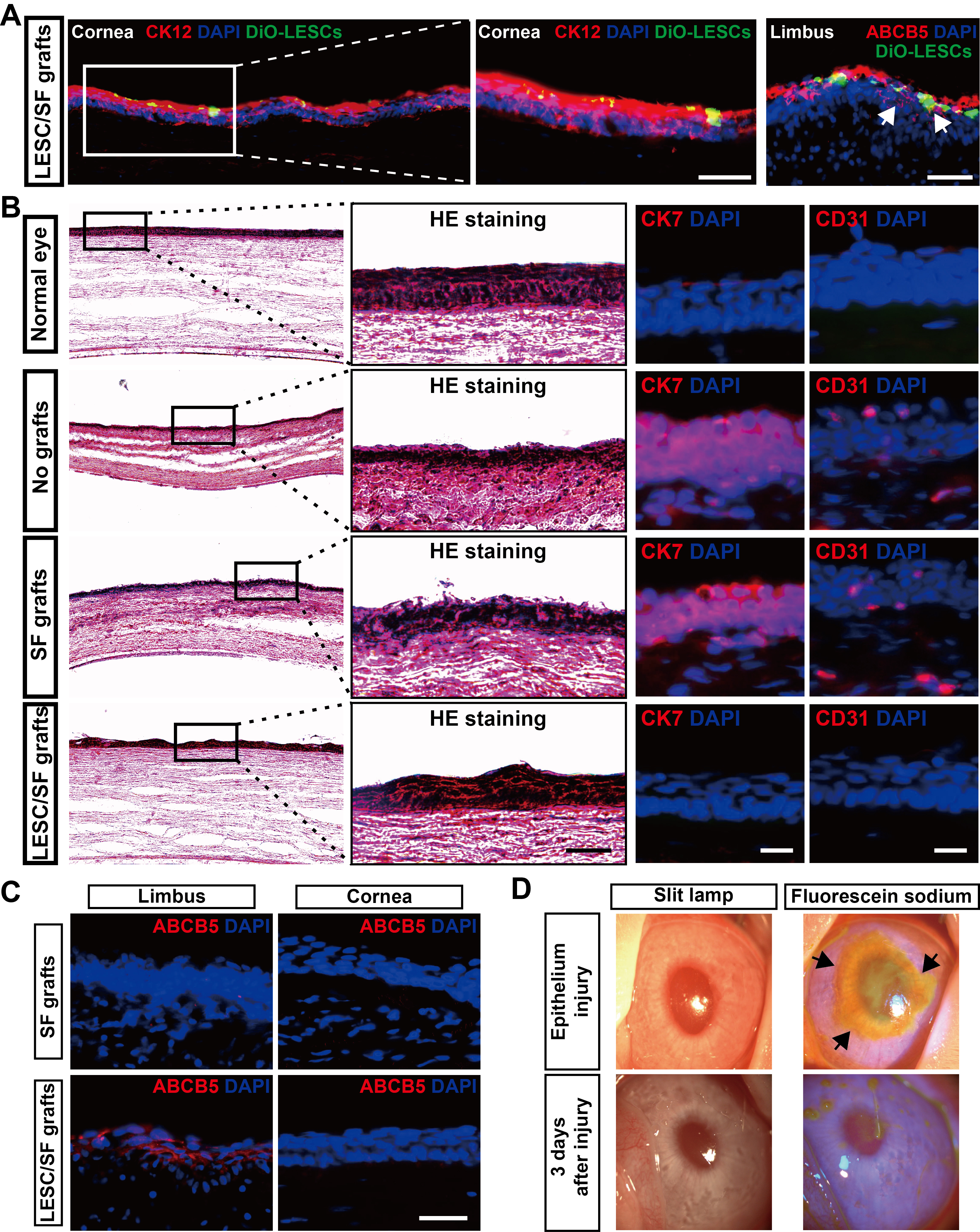

Supplement: Supplementary file 3 — Is Figure S3 showing restoration of LSCD and repopulated limbus by LESC/SF graft transplantation. (A) Rabbit corneas 2 months after LESC/SF graft transplantation (left panel, corneal epithelial cells marker CK12 staining; middle panel, enlarged pictures of the framed area; right panel, proposed LESCs marker ABCB5 staining in the limbus). Before LESC/SF graft transplantation, LESCs were labeled by DiO (DiO-LESCs, green) to trace these donor LESCs. More transplanted LESCs survived in the limbal region, but not in the cornea. Arrows point to ABCB5+ LESCs in the limbus. (B) Rabbit corneas 4 months after transplantation (left panels, HE staining; middle panels, enlarged pictures of the framed area; right panels, conjunctival epithelial cells marker CK7 staining and vascular endothelial cells marker CD31 staining). Normal corneas showed typical corneal epithelium. Corneas from no grafts (LSCD model) and SF grafts groups showed epithelial conjunctivalization and new blood vessels. Corneas from LESC/SF grafts group showed healed cornea surface without conjunctival epithelial cells and blood vessels. (C) LESC restoration in the limbus by LESC/SF grafts. ABCB5+ LESCs only existed in the limbal region but not in the cornea 4 months after LESC/SF transplantation, indicating that stem cell niche in the limbus was favorable for transplanted LESC survival and growth. (D) Repair of injured corneal epithelium once again. Top panels, regenerated corneal epithelium 4 months after initial LESC/SF graft transplantations was scraped off and made a large corneal epithelium defect (arrows). Bottom panels, injured corneal epithelium restored once again within 3 days with healed epithelial defect. Scale bar, 50 μm. (JPG 7374 kb) [file 13287_2017_707_MOESM3_ESM.jpg]
